# Redefining Immune Dynamics in Acute Pancreatitis: The Protective Role of Galectin-3 Deletion and Treg Cell Enhancement

**DOI:** 10.3390/biom14060642

**Published:** 2024-05-30

**Authors:** Ivana Milivojcevic Bevc, Danijela Tasic-Uros, Bojana S. Stojanovic, Ivan Jovanovic, Milica Dimitrijevic Stojanovic, Nevena Gajovic, Milena Jurisevic, Gordana Radosavljevic, Jelena Pantic, Bojan Stojanovic

**Affiliations:** 1 City Medical Emergency Department, 11000 Belgrade, Serbia; ivana.bevc@yahoo.com (I.M.B.); tasicurosdanijela@gmail.com (D.T.-U.); 2 Center for Molecular Medicine and Stem Cell Research, Faculty of Medical Sciences, University of Kragujevac, 34000 Kragujevac, Serbia; ivanjovanovic77@gmail.com (I.J.); gajovicnevena@yahoo.com (N.G.); milenajm@yahoo.com (M.J.); perun.gr@gmail.com (G.R.); panticjelena55@gmail.com (J.P.); bojan.stojanovic01@gmail.com (B.S.); 3 Department of Pathophysiology, Faculty of Medical Sciences, University of Kragujevac, 34000 Kragujevac, Serbia; 4 Department of Pathology, Faculty of Medical Sciences, University of Kragujevac, 34000 Kragujevac, Serbia; 5 Department of Pharmacy, Faculty of Medical Sciences, University of Kragujevac, 34000 Kragujevac, Serbia; 6 Department of Surgery, Faculty of Medical Sciences, University of Kragujevac, 34000 Kragujevac, Serbia

**Keywords:** acute pancreatitis, Galectin-3, immune modulation, regulatory T cells, dendritic cells

## Abstract

Acute pancreatitis (AP) is a complex inflammatory condition that can lead to systemic inflammatory responses and multiple organ dysfunction. This study investigates the role of Galectin-3 (Gal-3), a β-galactoside-binding lectin, in modulating acquired immune responses in AP. Acute pancreatitis was induced by ligation of the bile-pancreatic duct in wild-type and Galectin-3-deficient C57BL/6 mice. We determined the phenotypic and molecular features of inflammatory cells, serum concentrations of amylase, pancreatic trypsin activity, and pancreatic and lung pathology. Galectin-3 deficiency decreased the total number of CD3^+^CD49^−^ T cells and CD4^+^ T helper cells, downregulated the production of inflammatory cytokine and IFN-γ, and increased the accumulation of IL-10-producing Foxp3^+^ T regulatory cells and regulatory CD4^+^ T cells in the pancreata of diseased animals. The deletion of Galectin-3 ameliorates acute pancreatitis characterized by lowering serum amylase concentration and pancreatic trypsin activity, and attenuating of the histopathology of the lung. These findings shed light on the role of Galectin-3 in acquired immune response in acute pancreatitis and identify Galectin-3 as an attractive target for investigation of the immunopathogenesis of disease and for consideration as a potential therapeutic target for patients with acute inflammatory disease of the pancreas.

## 1. Introduction

Acute pancreatitis (AP) represents an acute inflammatory disorder of the pancreatic tissue that may not only affect nearby regional tissues but also extend its impact to remote organ systems, potentially resulting in systemic inflammation [1,2]. AP stands as a prevalent gastrointestinal reason for hospital admissions, with an annual incidence ranging from 20 to 40 cases per 100,000 individuals [3]. The primary etiologies of AP include alcohol abuse and obstruction caused by gallstones. Other, less prevalent, etiological factors comprise trauma, surgical interventions, overeating, metabolic disorders, and infections [4].

The initial phase in the pathogenesis of AP involves injury to acinar cells, precipitated by premature activation of enzymes. This cellular damage triggers the activation of innate immune cells and initiates a complex cascade of cytokines [5]. Subsequent to this initial response, the adaptive immune system is engaged through various mediators [6]. In severe cases of AP, designated as severe acute pancreatitis (SAP), the pathology is exacerbated by an overwhelming activation of innate immune cells, particularly macrophages [7]. T cells play a pivotal role in regulating the activation of macrophages, thereby modulating systemic immune responses [8]. Moreover, the extent of immune cell activation and the resultant secretion of inflammatory mediators correlate directly with the severity of AP. Thus, it is imperative to explore the factors that influence the activation of adaptive immune cells within the context of AP [6,9].

Galectin-3 (Gal-3) is a multifaceted molecule consisting of a C-terminal carbohydrate-recognition domain and an N-terminal, or lectin-like domain, which are critical for its diverse functions [10]. Gal-3 is instrumental in regulating key biological processes including regeneration, cell migration, and immune responses [11]. It is localized on the cell surface, within the cytoplasm and nucleus, and may also be secreted into extracellular spaces and the systemic circulation [12]. The role of Gal-3 in inflammation appears to be dualistic, exhibiting pro-inflammatory or anti-inflammatory effects depending on its extracellular or intracellular localization [13]. Recent studies in animal models have elucidated that Gal-3 plays a significant role in modulating immune responses by suppressing T cell activities [11]. Specifically, extracellular Gal-3 influences the formation of the immunological synapse, thereby attenuating T cell activation. Additionally, intracellular Gal-3 interacts with various membrane lipids and proteins, influencing endocytosis and signaling through the T cell receptor [14].

In a previous study [15], we observed that the absence of Gal-3 led to a diminished pro-inflammatory environment following the induction of AP. This was characterized by a reduced influx of N1 neutrophils, inflammatory macrophages, and dendritic cells, alongside decreased secretion of TNF-α and IL-1β. Collectively, these findings suggest that the genetic deletion of Gal-3 alleviates AP by impairing the early infiltration of neutrophils and pro-inflammatory mononuclear cells of the innate immune system. This effect is mediated through the interaction between Gal-3 and Toll-like receptor 4 (TLR4) [16]. Moreover, a significant role of Gal-3 in the onset and progression of pancreatic ductal adenocarcinoma (PDAC) has also been described [17]. To date, there is no data regarding the impact of Gal-3 deletion on acquired immunity during AP development. As our understanding of the immune mechanisms in AP advances, manipulating the immune response may offer a viable therapeutic strategy for treating patients with severe forms of the disease.

In this study, we demonstrate that the induction of AP via ligation of the bile-pancreatic duct results in a diminished influx of T cells into the pancreas, a reduced accumulation of interferon-gamma (IFN-γ)-producing T cells, and an increased accumulation of interleukin-10 (IL-10)-producing Foxp3^+^ regulatory T cells, as well as CD4^+^ regulatory cells following AP induction. Significantly, the influx of tolerogenic dendritic cells that produce the anti-inflammatory cytokine IL-10 is enhanced in mice with a genetic deletion of Gal-3. Furthermore, these findings indicate that the inflammatory dysregulation observed in mice with a genetic deletion of Gal-3 leads to a diminished extent of injury to both the pancreas and lungs following AP induction.

## 2. Materials and Methods

### 2.1. Animals

Animals utilized in this research comprised Galectin-3-deficient (Gal-3^−/−^) and wild-type (WT) C57BL/6 male mice, aged 6–8 weeks, on a C57BL/6 genetic background. The Gal-3 gene was targeted for disruption as previously described [18]. Both Gal-3^−/−^ and WT C57BL/6 breeding pairs from the same substrain were maintained under standard laboratory conditions at the Faculty of Medical Sciences, University of Kragujevac, Serbia. These conditions included a temperature of 22 ± 2 °C, relative humidity of 51 ± 5%, and a 12 h light–dark cycle. The animal study protocol was approved by the Ethics Committee for the Protection of the Welfare of Experimental Animals, Faculty of Medical Sciences, University of Kragujevac, under approval code 01-5268 on 26 April 2016, ensuring compliance with applicable guidelines and regulations.

### 2.2. Induction of Acute Pancreatitis by Bile-Pancreatic Duct (BPD) Ligation

Acute pancreatitis was induced in male mice via BPD ligation to simulate severe gallstone-induced pancreatitis, following the methodology outlined by Samuel et al. [19], with minor modifications [15]. Mice were anesthetized using a Ketamine/Xylazine cocktail administered intraperitoneally at doses of 70 mg/kg and 10 mg/kg, respectively. Surgical procedures were conducted under aseptic conditions. A midline laparotomy was performed, during which the BPD was dissected and ligated near its junction with the duodenum. In sham-operated control groups, the BPD was dissected but not ligated. The experimental groups were as follows: Gal-3^−/−^ mice with BPD ligation, wild-type (WT) mice with BPD ligation, Gal-3^−/−^ mice with sham operation, and WT mice with sham operation. Postoperative care included administration of Tramadol for analgesia at a dose of 20 mg/kg subcutaneously twice daily, and mice had ad libitum access to food and water. For assessments of disease severity and cellular infiltration analysis, mice were euthanized 72 h after the surgery. Plasma samples were collected and stored at −20 °C for further analysis. The pancreas was meticulously dissected from its attachments to the stomach, duodenum, spleen, and surrounding lymph nodes for subsequent examination.

### 2.3. Histological Evaluation of Pancreatic, Lung, and Kidney Injury

Portions of the pancreas, lung, and kidney tissues from all animal groups were fixed in 4% buffered formalin overnight, embedded in paraffin, and sectioned at 5 μm thickness. These sections were stained with hematoxylin and eosin (HE) and analyzed in a blinded fashion by two independent observers. The scoring system employed for pancreatic evaluation, as previously described, encompasses assessments of the extent of edema, leukocyte infiltration, necrosis, and hemorrhage [20]. Lung and kidney injuries were similarly assessed, with lung injury graded on alveolar congestion, hemorrhage, neutrophil infiltration into the airspace or vessel wall, and the thickness of the alveolar wall/hyaline membrane formation, as previously described [21]. The severity of kidney injury was scored using a methodology previously outlined [22]. Images from all tissues were captured using a light microscope (BX51; Olympus, Tokyo, Japan) equipped with a digital camera.

### 2.4. Levels of Amylase

Serum amylase levels were quantified in both experimental and control groups utilizing a colorimetric assay with a commercially available kit from Abcam, Cambridge, UK, according to the manufacturer’s instructions. The assay operates through a two-step reaction mechanism. Initially, α-amylase acts on the substrate ethylidene-pNP-G7 to generate smaller fragments. Subsequently, these fragments undergo further modification by α-glucosidase, resulting in the release of p-nitrophenol, which can be measured colorimetrically. The absorbance of these compounds is determined at an optical density (OD) of 405 nm. Measurements are taken in kinetic mode, at intervals of 2 to 3 min over a period of 30 to 60 min, at a temperature of 25 °C, and under conditions shielded from light [23].

### 2.5. Trypsin Activity

Trypsin activity was assessed using the trypsin colorimetric assay (ab102531, Abcam, Cambridge, UK) as per the specified protocol [24]. Briefly, pancreatic tissue homogenates were prepared in an ice-cold buffer. The activity of trypsin in these homogenates was quantitatively determined by its ability to cleave a specific substrate, resulting in the release of p-nitroaniline. This product was subsequently detected at an optical density (OD) of 405 nm.

### 2.6. Isolation of Leukocytes from Pancreata

Leukocytes were isolated from pancreatic tissues using a collagenase digestion method, as previously detailed [25]. Post-digestion, the tissues were gently disaggregated through a 40 μm cell-strainer nylon mesh using the plunger of a sterile syringe. The cells were then collected by centrifugation and resuspended in red blood cell lysing buffer for 5 min to remove erythrocytes. Finally, the isolated cells were resuspended in RPMI medium (Sigma Aldrich, St. Louis, MO, USA) supplemented with 10% fetal calf serum, preparing them for subsequent flow cytometric analysis.

### 2.7. Flow Cytometry

For flow cytometric analysis, isolated cells were labeled with an array of monoclonal antibodies: anti-mouse TNF-α, CTLA-4, IFN-γ, and IL-10 antibodies conjugated with fluorescein isothiocyanate (FITC); anti-mouse CD4 and CD49 antibodies conjugated with peridinin chlorophyll protein (PerCP); and anti-mouse CD3, IL-10, and CD49 antibodies conjugated with allophycocyanin (APC), all sourced from BD Bioscience, Franklin Lakes, NJ. For intracellular staining, cells were activated using phorbol myristate acetate (PMA) at a concentration of 50 ng/mL and ionomycin at 500 ng/mL (Sigma-Aldrich, St. Louis, MO, USA), supplemented with GolgiStop (BD Biosciences, Franklin Lakes, NJ, USA) for 5 h at 37 °C. Post-activation, cells were stained using the Cytofix/Cytoperm kit (BD Biosciences) in accordance with the manufacturer’s guidelines. Flow cytometric analysis was performed on a BD Biosciences FACS Calibur system and data were analyzed using FlowJo v10.7.2. (Tree Star, Ashland, OR, USA).

### 2.8. Statistical Analysis

All data are presented as mean ± SEM (standard error of the mean). The normality of the data distribution was assessed using the Kolmogorov–Smirnov test. Based on the results of this test, statistical comparisons between groups were performed using either the two-tailed Student’s *t*-test for normally distributed data or the nonparametric Mann–Whitney rank sum test for data not following a normal distribution. A *p*-value of less than 0.05 was considered to indicate statistical significance. All statistical analyses were conducted using the SPSS version 20 software package.

## 3. Results

### 3.1. Reduced Pancreatic Damage in Galectin-3-Deficient Mice Post-BPD Ligation

**Figure 1:**
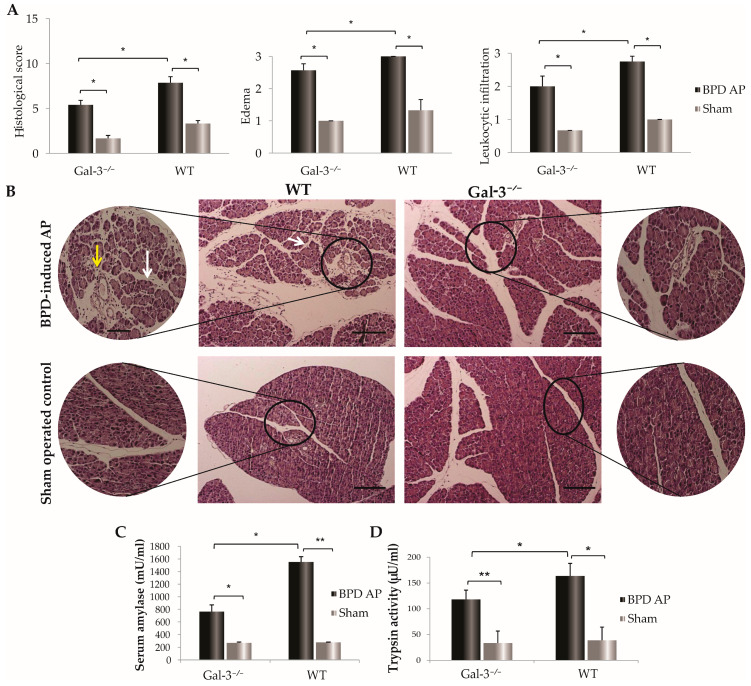
Impact of Gal-3 deletion on pancreatic injury in AP. (**A**): Total histological score of AP, sub-scores of edema and inflammatory cell. Data are shown as mean + SEM of 14 mice per group and are representative of two experiments; * *p* < 0.05, statistical significance was determined by Mann–Whitney U-test. (**B**): Photomicrographs of representative HE-stained mouse pancreas 12 h after BPD-induced AP. Massive interacinar edema (white arrows) and infiltration of inflammatory cells (yellow arrows) were detected in WT mice with BPD-induced AP in comparison to Gal-3^−/−^ mice with AP where only mild interlobular edema was present. Photomicrographs of representative HE-stained mouse pancreas from sham-operated control group that were without signs of pancreatic injury. Scale bar: 200 μm. Images are representative of two independent experiments. (**C**): Serum levels of amylase. Data are shown as mean + SEM of 12 mice per group and are representative of two independent experiments; * *p* < 0.05, ** *p* < 0.001, two-tailed unpaired Student’s *t*-test. (**D**): The intrapancreatic trypsin activity. Data are shown as mean + SEM of 12 mice per group and are representative of two independent experiments; * *p* < 0.05, ** *p* < 0.001, two-tailed unpaired Student’s *t*-test.

Seventy-two hours following the bile-pancreatic duct ligation, histological evaluation of the hematoxylin and eosin (HE)-stained sections of the pancreas revealed significantly lower total acute pancreatitis histological scores in Gal-3^−/−^ mice compared to WT controls. As shown in Figure 1A, Gal-3^−/−^ mice with acute pancreatitis exhibited lower total histological scores, with decreased sub-scores for edema and leukocyte infiltration than WT mice (*p* < 0.05, Figure 1A). Representative photomicrographs demonstrated widespread areas of intralobular and interacinar edema, along with significant infiltration of inflammatory cells in WT mice, in contrast to the milder infiltration observed in the pancreatic sections of Gal-3^−/−^ mice (Figure 1B). Additionally, 72 h post-ligation, serum amylase levels were significantly lower in Gal-3^−/−^ mice with AP than in WT mice (M ± SEM; 765.04 ± 83.04 vs. 1552.17 ± 105.64; *p* < 0.05) (Figure 1C). A significant difference was also noted in pancreatic trypsin activity between the Gal-3^−/−^ and WT mice (M ± SEM; 118.10 ± 18.11 vs. 163.72 ± 24.38; *p* < 0.05) (Figure 1D).

### 3.2. Galectin-3 Deficiency and Its Protective Effects against Lung Injury in Acute Pancreatitis

**Figure 2:**
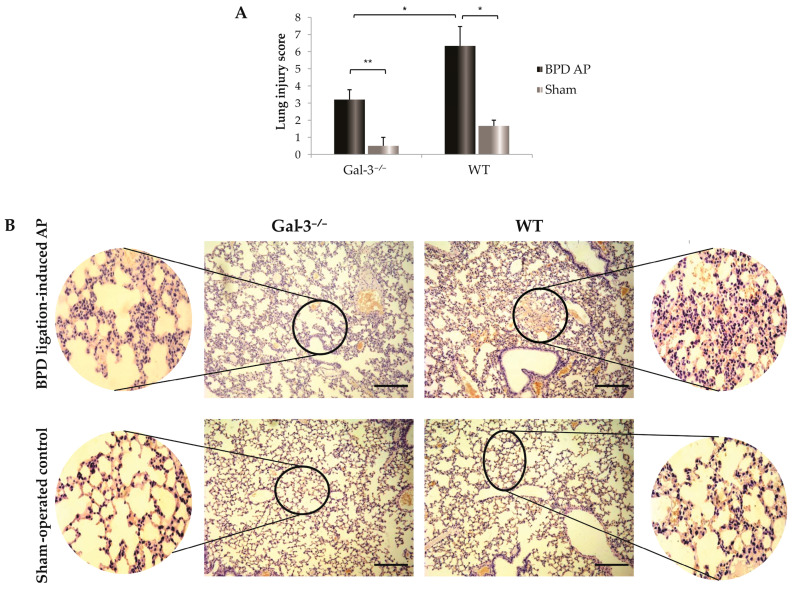
Galectin-3 deletion and its effects on the lung. (**A**): Total histological score of lung injury. Data are shown as mean + SEM of 14 mice per group and are representative of two independent experiments; * *p* < 0.05, ** *p* < 0.001, statistical significance was determined by Mann–Whitney U-test. (**B**): Photomicrographs of representative HE-stained mouse lung after induction of AP by BPD ligation. The lungs of the WT mice with AP showed significant interstitial edema, patchy hemorrhage, thickened alveolar interstitium, and infiltration of inflammatory cells compared to Gal-3^−/−^ mice with AP. The lungs of the sham-operated controls showed no significant swelling, inflammation, or infiltration. Scale bar: 200 μm. Images are representative of two independent experiments.

As acute pancreatitis can also impact lung and kidney tissues, we examined inflammatory changes in these organs. Within 72 h after bile-pancreatic duct ligation, no significant alterations were observed in the kidneys. However, significant changes were noted in the lungs, as evidenced by histological examination. Specifically, lung tissue damage was characterized by a significantly higher influx of leukocytes in the lungs of WT mice, along with hemorrhage—features that were not observed in Gal-3^−/−^ mice (Figure 2A,B).

### 3.3. Impact of Galectin-3 Deletion on T Cell Dynamics in Acute Pancreatitis

**Figure 3:**
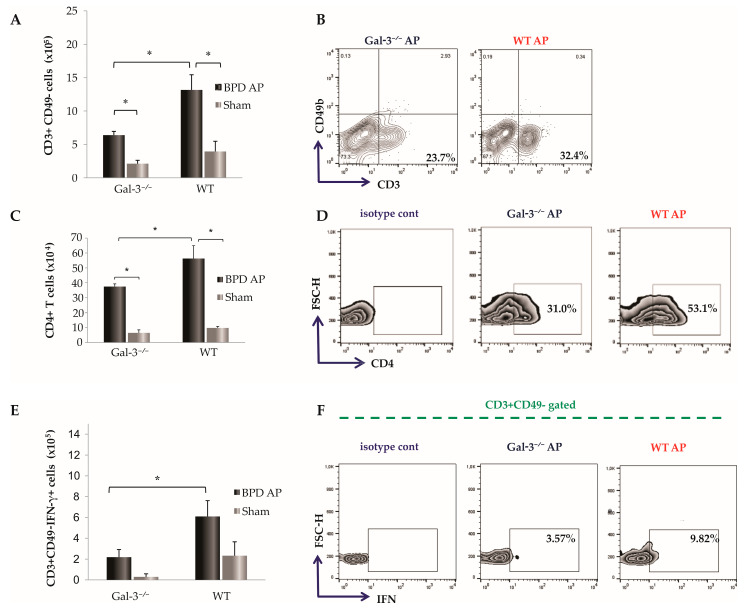
T cells with pro-inflammatory phenotype are attenuated in Gal-3-deficient mice after induction of disease by BPD ligation. The total number and representative FACS plots displaying the frequency of CD3^+^CD49^−^ cells (**A**,**B**), CD4^+^ cells (**C**,**D**), and IFN-γ-producing CD3^+^CD49^−^ cells (**E**,**F**) derived from the pancreas. Data are shown as mean + SEM of 12 mice per group and are pooled from two independent experiments; * *p* < 0.05, two-tailed unpaired Student’s *t*-test.

Seventy-two hours after BPD ligation, we examined the cellular makeup of leukocyte infiltration in the pancreatic tissue of Gal-3^−/−^ mice and WT mice, both in diseased and sham-operated controls. As previously reported [15], Gal-3 genetic deletion led to a significantly reduced total number of infiltrating leukocytes in pancreatic tissue. Additionally, the total number of CD3^+^CD49^−^ T cells was significantly higher in the pancreata of diseased WT mice compared to Gal-3^−/−^ mice (*p* < 0.05, Figure 3A). The percentage of CD3^+^CD49^−^ T cells was also significantly increased in WT mice compared to Gal-3^−/−^ mice with AP (33.68 ± 8.35 vs. 23.95 ± 3.50; *p* < 0.05; illustrated in Figure 3B). Correspondingly, the total number of CD4^+^ cells was significantly decreased in Gal-3^−/−^ mice seventy-two hours after induction of AP (*p* < 0.05, Figure 3C), and the percentage of CD4^+^ cells was notably diminished in diseased Gal-3^−/−^ mice compared to WT mice (46.53 ± 6.52 vs. 30.32 ± 1.84; *p* < 0.05; Figure 3D). Intracellular staining of T cells revealed that Gal-3^−/−^ mice with AP had significantly lower percentages and total numbers of IFN-γ-producing T cells compared to WT mice with AP (2.72 ± 1.04 vs. 4.94 ± 0.56; *p* < 0.05; Figure 3E,F). No differences were found between the groups in terms of TNF-α-producing T cells. When observing changes in the number of examined cells compared to sham-operated controls, there were no statistically significant differences between Gal-3^−/−^ and WT mice.

### 3.4. Pronounced Infiltration of Regulatory T Cells in Pancreatic Tissue of Diseased Gal-3-Deficient Mice

**Figure 4:**
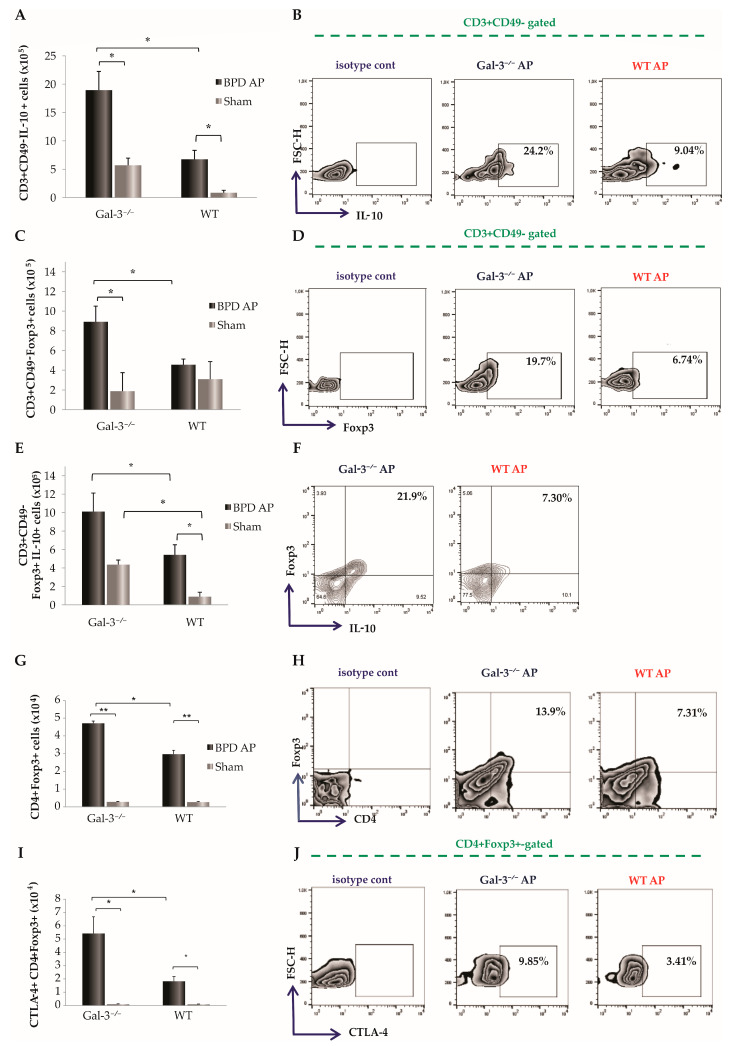
Gal-3 deletion alters T cell population in AP favoring influx of T regulatory cells and IL-10 production. The total number and representative FACS plots presenting the percentage of IL-10-producing (**A**,**B**), Foxp3-expressing CD49b^−^CD3^+^ cells (**C**,**D**), CD49b^−^CD3^+^ cells expressing Foxp3 and containing IL-10 (**E**,**F**), Foxp3-expressing CD4^+^ cells (**G**,**H**), and CD4^+^cells expressing Foxp3 and CTLA-4 (**I**,**J**). Data are shown as mean + SEM of 12 mice per group and are pooled from two independent experiments; * *p* < 0.05, ** *p* < 0.001, two-tailed unpaired Student’s *t*-test.

In order to explore the impact of Gal-3 deletion on inflammatory responses in pancreatic tissue post-acute pancreatitis induction, we analyzed the quantity and functional phenotype of regulatory T cells (Tregs) in the pancreata of naïve Gal-3^−/−^ and WT mice. Notably, concurrent with a decrease in the infiltration of IFN-γ-producing T cells, the number of CD3^+^CD49^−^ T cells producing IL-10 was significantly elevated in the Gal-3^−/−^ mice compared to the WT group (*p* < 0.05, Figure 4A). Furthermore, the frequency of IL-10-producing CD3^+^CD49^−^ T cells in the pancreata significantly increased in Gal-3^−/−^ mice following AP induction by BPD ligation (22.29 ± 4.12 vs. 8.07 ± 1.64; *p* < 0.05; Figure 4B). The number of these cells was also higher in sham-operated Gal-3^−/−^ mice compared to sham-operated WT mice, although the difference did not reach statistical significance. Additionally, both the percentage and total number of forkhead box P3 (Foxp3)-expressing T cells were markedly higher in the diseased Gal-3^−/−^ mice (11.23 ± 2.83 vs. 5.03 ± 0.47; *p* < 0.05; Figure 4C,D). The percentage and total count of Foxp3-expressing T cells that also secreted IL-10 were significantly increased in Gal-3^−/−^ mice with AP compared to WT mice with AP (12.07 ± 2.71 vs. 5.40 ± 1.23; *p* < 0.05; Figure 4E,F). In the sham-operated controls, the number of Foxp3-expressing T cells that also secreted IL-10 was also higher in Gal-3^−/−^ mice compared to WT mice. However, when compared to diseased animals, the number of examined cells was significantly lower in the sham-operated controls (Figure 4E). 

The investigation further extended to the influx of regulatory CD4^+^ cells. By 72 h post-BPD ligation, flow cytometric analysis of mononuclear cells isolated from the pancreatic tissue indicated a significant augmentation in the number of CD4^+^ cells expressing Foxp3 in Gal-3 deletion mice (*p* < 0.05, Figure 4G). Compared to diseased WT mice, the percentages of Foxp3-expressing CD4^+^ cells were significantly elevated in Gal3^−/−^ mice with AP (13.10 ± 0.90 vs. 7.80 ± 0.90; *p* < 0.05; Figure 4H). We also examined the number and relative percentage of Foxp3-expressing CD4^+^ cells that also express the CTLA-4 molecule, which plays a crucial role in establishing immune homeostasis [26]. The total number and frequency of CTLA-4-expressing CD4^+^Foxp3^+^ cells were significantly higher in the pancreas of Gal-3^−/−^ mice with BPD ligation-induced AP compared to WT mice (*p* < 0.05; Figure 4I,J).

### 3.5. Influence of Galectin-3 Deletion on Tolerogenic Dendritic Cell Infiltration in the Pancreas

**Figure 5:**
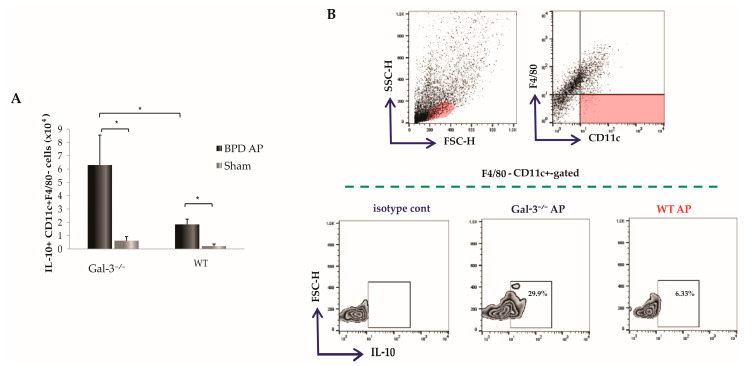
Galectin-3 deletion increases influx of tolerogenic phenotypes of dendritic cells. The total number (**A**) and representative FACS plots (**B**) presenting the percentage of IL-10-producing F4/80^−^CD11c^+^ cells. Data are shown as mean + SEM of 12 mice per group and are pooled from two independent experiments; * *p* < 0.05, two-tailed unpaired Student’s *t*-test.

Tolerogenic dendritic cells (DCs) are integral to the immunoregulatory environment, chiefly due to their capacity to produce anti-inflammatory cytokines such as IL-10, which facilitate the differentiation of naïve T cells into Foxp3^+^ regulatory T cells [27]. To explore the role of Gal-3 in modulating this cell population, we examined the effects of Gal-3 deletion on the presence of tolerogenic DCs in the pancreas post-injury. Intracellular staining demonstrated that mice lacking Gal-3 with induced AP had a significantly increased total number of IL-10-producing F4/80^−^CD11c^+^ DCs (*p* < 0.05, Figure 5A). Additionally, the proportion of IL-10-producing F4/80^−^CD11c^+^ DCs was significantly elevated 72 h following AP induction in Gal-3^−/−^ mice (25.24 ± 5.45 vs. 6.11 ± 1.00; *p* < 0.05; Figure 5B).

## 4. Discussion

In this study, we present evidence that Gal-3 deficiency confers a significant protective effect against the development of acute pancreatitis. Our findings indicate that the genetic deletion of Gal-3 markedly reduces the extent of inflammatory injury in both the pancreas and lungs. This reduction is mediated through decreased infiltration of T cells and CD4^+^ T cells within the pancreatic tissue, a lowered production of the pro-inflammatory cytokine IFN-γ, and an enhanced activation of regulatory T cells. The latter effect is facilitated by an increased influx of tolerogenic dendritic cells, further substantiating the role of Gal-3 as a key modulator of immune responses in the context of AP.

While it is anticipated that acquired immunity will develop during the later stages of disease progression, in the initial days, the key role is played by innate immunity, as we have previously published [10]. Despite these expectations, our current findings, along with those from similar studies such as Glaubitz J et al. [28], indicate that a developed acquired immune response is evident as early as the third day post-disease induction. This early activation of acquired immunity prompted us to further explore the effector cells of this immune response, particularly focusing on mechanisms that remain undescribed in the existing literature. The results of this investigation provide new insights into the complex dynamics of immune responses during the early phase of the disease, challenging traditional timelines associated with immune activation and offering new avenues for therapeutic intervention.

### 4.1. The Role of Galectin-3 in Modulating Inflammation and Organ Injury in Acute Pancreatitis

Acute pancreatitis is a common clinical emergency that frequently leads to multi-organ damage. This condition is characterized by the premature activation of digestive enzymes, resulting in autodigestion, inflammation, edema, bleeding, and even necrosis of the pancreas or surrounding adipose tissue [29]. Our findings demonstrate that Gal-3 is a critical immunomodulatory molecule in the development of pancreatic inflammation during AP. Specifically, the deletion of Gal-3 is associated with reduced inflammation, edema, and leukocyte infiltration, consistent with our previously published results (Figure 1A,B) [15]. Furthermore, a lower degree of pancreatic damage in Gal-3-deficient mice is characterized by statistically reduced trypsin activity in pancreatic parenchyma and lower serum amylase activity (Figure 1C,D).

The lungs are often the most directly affected extra-pancreatic organ in AP, contributing to lung injury, which is a principal cause of early mortality in AP patients, with a mortality rate of approximately 60% [30]. Pathological changes typical of acute lung injury include hyperosmotic pulmonary edema, increased alveolar fluid levels, and fields of hemorrhage within the lung parenchyma, as we have demonstrated in Figure 2 [31]. The generation of inflammatory mediators such as cytokines, chemokines, and reactive oxygen species (ROS), primarily by macrophages and neutrophils, underpins the mechanism of AP-induced lung injury [32]. Our data indicate that Gal-3 influences the activation and increased influx of pro-inflammatory M1 macrophages and N1 neutrophils [15]. Therefore, in the context of Gal-3 deficiency, there is a corresponding reduction in lung damage (Figure 2). Besides the direct effects on the activation of cells involved in innate immunity, it raises the question of whether Gal-3 also indirectly influences their activation through the modulation of adaptive immune activity.

### 4.2. Impact of T Cell Dynamics and Galectin-3 Deficiency on Acute Pancreatitis

T cells are crucial in the development of the systemic immune inflammatory response observed in AP [6,9]. It has been noted that while T cells decrease in circulation, there is an increased infiltration from blood vessels to the injury site adjacent to pancreatic acini during AP [33]. Experimental evidence suggests that the genetic depletion of T cells significantly attenuates the severity of experimental AP [34,35,36]. In our studies, Gal-3-deficient mice exhibited a markedly reduced infiltration of CD3^+^ T cells in the pancreas following BPD ligation-induced AP (Figure 3A). Gal-3 is known to facilitate leukocyte recruitment and may function as an adhesion molecule in inflammatory exudates [37]. A previous study also demonstrated that Gal-3 deficiency led to a significantly reduced number of T cells in the liver in experimental models of fulminant hepatitis [11].

Reports indicate that sensitized CD4^+^ T helper cells migrate to inflamed tissues, leading to a significant increase in CD4^+^ T-cell numbers in the pancreas during AP [38]. Additionally, an increased number of infiltrating T cells in the inflamed pancreas exacerbates local damage through the production of a plethora of pro-inflammatory cytokines [38]. In our research, we observed a significantly lower number of CD4^+^ T cells in the pancreata of mice with genetic deletion of Gal-3 (Figure 3C). This finding underscores the modulatory role of Gal-3 in T-cell dynamics and the inflammatory cascade in AP.

### 4.3. Impact of Galectin-3 Deficiency on T Cell Activation and IFN-γ Production in Acute Pancreatitis

During the development of AP, there is an early activation of T cells, which leads to the production of cytokines characteristic of the Th1 response, such as interferon-gamma (IFN-γ) [9]. Subsequently, these activated T cells contribute to the activation of innate immune cells, including macrophages and neutrophils [39]. Our findings reveal that Gal-3-deficient mice exhibit a reduced number of IFN-γ-positive T cells following AP induction (Figure 3E). Prior studies have highlighted that IFN-γ levels are elevated in the serum of patients with AP, where it plays a pivotal role in macrophage activation, thereby exacerbating inflammation and contributing to pancreatic necrosis [40,41].

### 4.4. Regulatory Influence of Galectin-3 Deficiency on Tregs Dynamics in Acute Pancreatitis

The interplay between pro-inflammatory and anti-inflammatory cytokine milieus critically influences the progression of AP, especially in its severe form (SAP) [42]. In this context, regulatory T cells (Tregs) play a pivotal role in modulating the immune response, effectively controlling and potentially depleting the inflammatory reactions that exacerbate the condition. Studies have demonstrated that Tregs can significantly downregulate pancreatic inflammation and thereby reduce mortality in models of severe AP [43]. Our research contributes to this body of knowledge by demonstrating a marked increase in the total number of Foxp3-expressing Tregs in Gal-3-deficient mice following the induction of AP (Figure 4F). This suggests a protective role of Gal-3 deficiency in modulating the inflammatory response through Tregs (Figure 4F).

Interestingly, in contrast to the increased Treg count, the production of IL-10, a key anti-inflammatory cytokine produced by Tregs, was significantly lower in WT mice (Figure 4E,F). Additionally, regulatory CD4^+^ T helper cells that express both Foxp3 and CTLA-4 were significantly more infiltrated in the pancreatic tissue of Gal-3-deficient mice post-AP induction, reinforcing the notion that Gal-3 deficiency enhances the anti-inflammatory regulatory capacity of Tregs. These findings align with prior research indicating that the deletion of Gal-3 leads to a significant increase in the percentage of CD4^+^CD25^+^Foxp3^+^ Treg cells in models of fulminant hepatitis [44]. Furthermore, Treg cells from Gal-3 knockout mice have been shown to produce a higher quantity of IL-10 compared to WT Treg cells, thereby affecting susceptibility to Leishmania major infection [45].

### 4.5. Impact of Galectin-3 Deficiency on Tolerogenic DCs and Treg Infiltration in AP

In recent years, our understanding of dendritic cells (DCs) in both the initiation and regulation of immune responses has expanded significantly. In the context of acute pancreatitis (AP), DCs display a dichotomous function; they are capable of both promoting and suppressing the inflammatory response [42,46]. Particularly, DCs with a tolerogenic phenotype, characterized by their production of the anti-inflammatory cytokine IL-10, are crucial in modulating the immune response [47]. These cells drive the increase in infiltration of Tregs in inflamed tissue, thereby aiding in the resolution of inflammation [48]. 

Moreover, it has been reported that tolerogenic DCs play a pivotal role in restraining disease progression [49]. Conversely, their systemic depletion results in severe acinar cell damage, increased dysfunction of the pancreas, and heightened mortality [49]. Additionally, these DCs influence the adaptive immune system and contribute to the development of chronic pancreatitis [33]. They facilitate the resolution of ongoing immune responses through mechanisms involving cell–cell contact and cytokine production, particularly IL-10, which is essential in controlling both effector and regulatory mechanisms critical to the pathology of inflammatory disorders [47].

**Figure 6:**
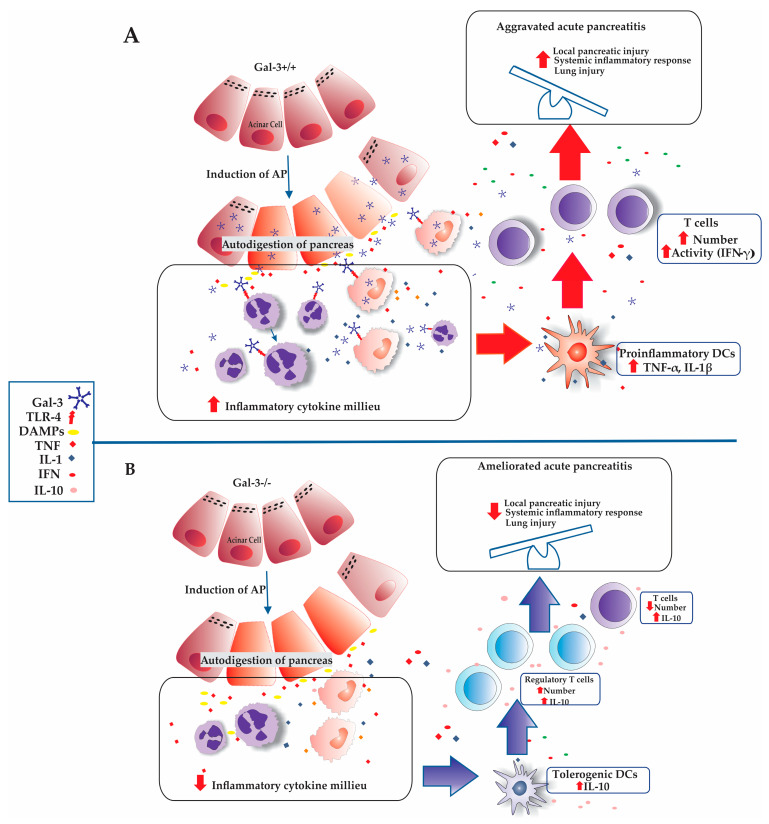
Galectin-3 deletion diminishes inflammatory cytokine milieu and promotes regulatory mechanisms of innate and acquired immunity. (**Panel A**): BPD ligation leads to autodigestion of acinar cells and consecutive release of alarmins and Galectin-3. Released Galectin-3 interacts with TLR-4 receptor on the proximate neutrophils and macrophages, promoting pro-inflammatory cytokine milieu and inducing development and predomination of pro-inflammatory DCs as well as effector inflammatory cells of acquired immunity and production of inflammatory cytokines. As a result derives severe inflammatory tissue damage. (**Panel B**): In absence of Galectin-3, activation of neutrophils and macrophages is attenuated, as well as production of pro-inflammatory cytokines, facilitating predominance of tolerogenic phenotype of dendritic cells leading to activation of regulatory Foxp3^+^ IL-10 producing cells. This microenvironment attenuates tissue destruction via immune mechanisms.

Furthermore, tolerogenic DCs are known to stimulate the induction and expansion of different subsets of regulatory lymphocytes, such as Foxp3^+^ Tregs [50]. As illustrated in Figure 5, the number and percentage of IL-10 producing DCs are significantly higher in the pancreata derived from Gal-3^−/−^ mice following AP induction by BPD ligation. This increase suggests a mechanism where the absence of Gal-3 and its interaction with TLR-4 reduce the infiltration of N1 neutrophils, inflammatory macrophages, and dendritic cells, thereby creating a less pronounced pro-inflammatory cytokine milieu [15,16]. This shift promotes the infiltration of DCs with a tolerogenic phenotype, which produce downregulatory cytokines, particularly IL-10, enhancing the influx of regulatory T lymphocytes into the pancreatic tissue of Gal-3^−/−^ mice (Figure 6).

The resultant increase in Treg infiltration and production of anti-inflammatory cytokines correlates with a reduced extent of local pancreatic tissue damage, a diminished systemic inflammatory response, and less severe lung damage in mice with Gal-3 deletion after AP induction (Figure 1 and Figure 2). Previous research has demonstrated that the lesser extent of pancreatic damage in mice lacking endogenous Gal-3 is characterized primarily by intralobular and inter-acinar edema, as opposed to the widespread areas of necrosis, leukocyte infiltration, and severe edema observed in WT mice [15]. This aligns with the findings that Gal-3-deficient mice exhibit markedly lower levels of serum amylase and pancreatic trypsin activity compared to WT mice with AP (Figure 1A–D and Figure 2). Correspondingly, AP-induced lung histopathological damage was significantly reduced in Gal-3-deficient mice within 72 h post-BPD ligation, evidenced by mild interstitial edema, decreased thickening of alveolar interstitium, and reduced infiltration of inflammatory cells (Figure 2A,B).

### 4.6. Limitations and Future Directions

Despite the significant findings, our study has several limitations that need to be acknowledged. First, the study was conducted exclusively on a murine model, which may not fully replicate the complexity of acute pancreatitis in humans. Second, the induction of acute pancreatitis through bile-pancreatic duct ligation, while effective, may not encompass all etiological factors present in human cases of the disease. Third, the study focused primarily on the early immune response, particularly within the first 72 h post-induction, potentially overlooking longer-term immune dynamics and their impacts on disease progression and resolution. Additionally, the study did not assess potential therapeutic interventions or the effects of pharmacological modulation of Gal-3, which could provide insights into its viability as a therapeutic target.

Building on these limitations, several areas warrant further investigation to deepen our understanding and enhance the therapeutic potential of targeting Gal-3. Future research should focus on validating these findings in clinical settings to provide more direct evidence of Gal-3’s relevance to human disease. Examining the long-term effects of Gal-3 deficiency on disease progression and resolution will help elucidate the chronic implications of its modulation in acute pancreatitis.

Employing tissue-specific knockout models and organ-specific knockout mice will provide detailed insights into the role of Gal-3 in different tissues and its impact on disease pathogenesis and immune responses. Utilizing different experimental models that enable longer follow-up periods will be essential to study the late stages of the inflammatory response and the activity of the innate immune system.

Further exploration is needed to characterize the specific acquired immune mechanisms influenced by Gal-3. Detailed profiling of the effector cells involved, including different subsets of T cells and their cytokine profiles, will provide a comprehensive view of the immune landscape in acute pancreatitis. Investigating how Gal-3 influences the phenotype of acquired immune cells and its contribution to the development of chronic pancreatitis will be crucial for identifying potential therapeutic targets for chronic inflammatory diseases of the pancreas.

Investigating the molecular signaling pathways through which Gal-3 exerts its effects on immune cells will enhance our understanding of its role in inflammation and immunity. Evaluating the effects of pharmacological inhibitors of Gal-3 in animal models and clinical trials will be essential to determine its viability as a therapeutic target, assessing both efficacy and potential side effects.

By addressing these areas, future research can build on the findings of this study, advancing our knowledge of immune regulation in acute pancreatitis and potentially leading to novel therapeutic strategies.

## 5. Conclusions

In conclusion, our study provides compelling evidence that the genetic deletion of Galectin-3 markedly diminishes the severity of acute pancreatitis and its systemic complications. By attenuating the infiltration and activation of various immune cells within the pancreatic tissue, Gal-3 deletion leads to a significant reduction in local and systemic inflammation. The associated increase in regulatory T cell activity and the favorable shift in dendritic cell phenotypes towards a more tolerogenic state further highlight the potential of targeting Gal-3 as a therapeutic strategy. These findings not only deepen our understanding of the immunological mechanisms underpinning acute pancreatitis but also underscore the therapeutic potential of modulating Galectin-3 in mitigating the disease’s impact, paving the way for more effective treatments.

## Data Availability

The datasets generated and/or analyzed during the current study are available from the corresponding author on reasonable request.
